# Transdermal Delivery of 2-PAM as a Tool to Increase the Effectiveness of Traditional Treatment of Organophosphate Poisoning

**DOI:** 10.3390/ijms232314992

**Published:** 2022-11-30

**Authors:** Leysan Vasileva, Gulnara Gaynanova, Irina Zueva, Anna Lyubina, Syumbelya Amerhanova, Daina Buzyurova, Vasily Babaev, Alexandra Voloshina, Konstantin Petrov, Lucia Zakharova

**Affiliations:** Arbuzov Institute of Organic and Physical Chemistry, FRC Kazan Scientific Center, Russian Academy of Sciences, 420088 Kazan, Russia

**Keywords:** transfersome, 2-PAM, alkylpyrrolidinium bromide, organophosphate poisoning, transdermal drug delivery, acetylcholinesterase

## Abstract

For the first time, the efficacy of post-exposure treatment of organophosphate (OP) poisoning was increased by transdermal delivery of acetylcholinesterase (AChE) reactivator pyridine-2-aldoxime methochloride (2-PAM) as a preventive countermeasure. By selecting the optimal ratio of components, classical transfersomes (based on soybean phosphatidylcholine and Tween 20) and modified transfersomes (based on soybean phosphatidylcholine, Tween 20 and pyrrolidinium cationic surfactants with different hydrocarbon tail lengths) were obtained for 2-PAM encapsulation. Transfersomes modified with tetradecylpyrrolidinium bromide showed the best results in encapsulation efficiency and sustained release of 2-PAM from vesicles. Using Franz cells, it was found that the incorporation of surfactants into PC liposomes results in a more prolonged release of 2-PAM through the rat skin. Transfersomes containing 2-PAM, after exhaustive physical and chemical characterization, were embedded in a gel based on Carbopol^®^ 940. A significantly high degree of erythrocyte AChE reactivation (23 ± 7%) was shown for 2-PAM in unmodified transfersomes in vivo. Preliminary transdermal administration of 2-PAM 24 h before emergency post-exposure treatment of OP poisoning leads to an increase in the survival rate of rats from 55% to 90%.

## 1. Introduction

There is a wide range of organophosphorus compounds (OPs) that are highly toxic and are used as pesticides and warfare nerve agents [1,2,3]. Acute OP poisoning leads to inhibition of the enzyme acetylcholinesterase (AChE), respiratory muscle weakness and rapid death due to asphyxia [4]. It was evaluated that OP-based pesticides are responsible for up to 3 million cases of intoxications every year, with 200,000 of them being fatal [5,6]. Typically, in the treatment of acute OP poisoning, a combination of drugs is administered, such as an AChE reactivator (oxime), an antimuscarinic drug (e.g., atropine), and an anticonvulsant drug (e.g., diazepam) [3,7]. The efficiency of the standard therapy of acute OP poisoning in most cases is unsatisfactory. Among the main factors limiting the effectiveness of antidote therapy for OP poisoning is the extremely short period after poisoning, when the administration of antidotes can reduce mortality. However, if OP exposure is expected, prophylactic countermeasures may be taken to mitigate poisoning and increase the efficiency of standard therapy. Administered as a pretreatment, reversible AChE inhibitors bind to the enzyme, thereby protecting its active site from irreversible inhibition by OPs [8,9,10,11,12,13]. Another promising avenue for development of prophylactic countermeasures against poisoning with OPs is the use of transdermal patches containing oximes [12,13,14]. Transdermal drug administration is gaining popularity because this non-invasive method avoids first-pass hepatic metabolism and provides a continuous drug administration [15,16,17,18]. In particular, some basic level of oxime in the bloodstream can be maintained via transdermal delivery, which gains the time needed for the application of standard emergency therapy. Only a couple of studies from two research groups are known that demonstrated transdermal administration of 2-PAM and HI-6 (asoxime chloride). For the combination of 2-PAM with eserine against (±)-Anatoxin A poisoning, a drug-in-adhesive matrix-type transdermal patch was formed that did not cause skin irritation [14,19], did not have mutagenic properties [20], and was stable for 6 months [21], and a pharmacokinetic study in rabbits showed that plasma levels of both drugs after transdermal application of one patch were maintained for 72 h after its removal [22]. There are studies describing the production of patches for HI-6 called TRANSANT, which were clinically tested (including dermal sensitivity) and introduced into the Czech and Slovak army [12,13,23]. It is also possible to use the TRANSANT patch in combination with PANPAL (tablets with pyridostigmine, trihexyphenidyle and benactyzine).

**Figure 1:**
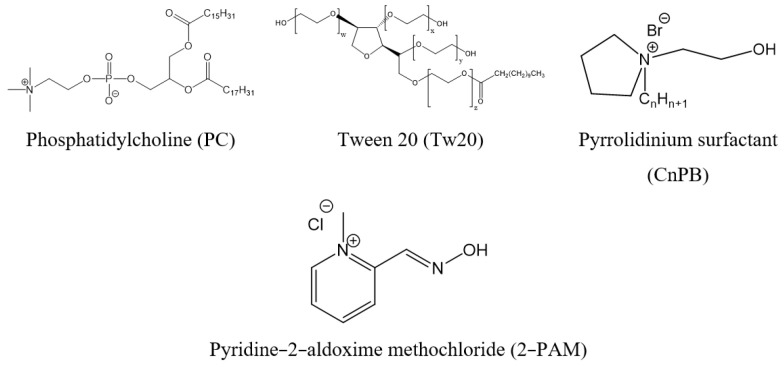
Structural formulas of compounds used.

To the best of our knowledge, there are no studies with the inclusion of AChE reactivators in liposomal systems for transdermal administration. Therefore, the aim of this work was to form ultradeformable liposomes (transfersomes) with 2-PAM. Transfersomes were obtained by selecting the optimal ratio of components in their classical (based on soybean phosphatidylcholine and Tween 20) and modified (based on soybean phosphatidylcholine, Tween 20 and pyrrolidinium surfactants) versions (Figure 1). The hydrodynamic diameter, electrokinetic potential, entrapment efficiency, and substrate release rate were evaluated for all systems. The penetration of 2-PAM-loaded transfersomes ex vivo was studied using Franz cells. Prior to experiments in rats, transfersomes with 2-PAM were included in a Carbopol-based gel to improve the rheological characteristics of the formulation. In vivo, the level of AChE reactivation was assessed at various time intervals, and pharmacokinetic studies made it possible to determine the concentration of 2-PAM in plasma after a single application of the developed gel.

## 2. Results

### 2.1. Transfersome Preparation and Characterization

In the current study, the classic transfersome composition (PC/Tw20) was modified with cationic surfactants bearing a pyrrolidinium head group with a hydroxyethyl moiety (C_n_PB, where *n* = 12, 14, and 16). To exclude the formation of micellar aggregates, the concentration of the C_n_PB in the composition of transfersomes was lower than their critical micelle concentration [24]. In all systems, the concentrations of PC and Tw20 were constant, and the concentration of the cationic surfactant was varied in the range of 0.2, 0.25, and 0.4 mM to obtain the following molar ratios of PC/Tw20/C_n_PB: 1/0.2/0.02, 1/0.2/0.025, and 1/0.2/0.04. According to the data obtained by dynamic light scattering, the hydrodynamic diameters of unmodified and modified transfersomes were within 120 nm. The polydispersity index (PdI) does not exceed 0.1 on average, which indicates that a monodisperse size distribution occurs (Table 1). There is also a trend towards an increase in the hydrodynamic diameter of vesicles with addition of C_n_PB to the composition of transfersomes. It should be noted that a similar trend is also observed in the case of PdI and zeta potential (ζ) values.

**Figure 2:**
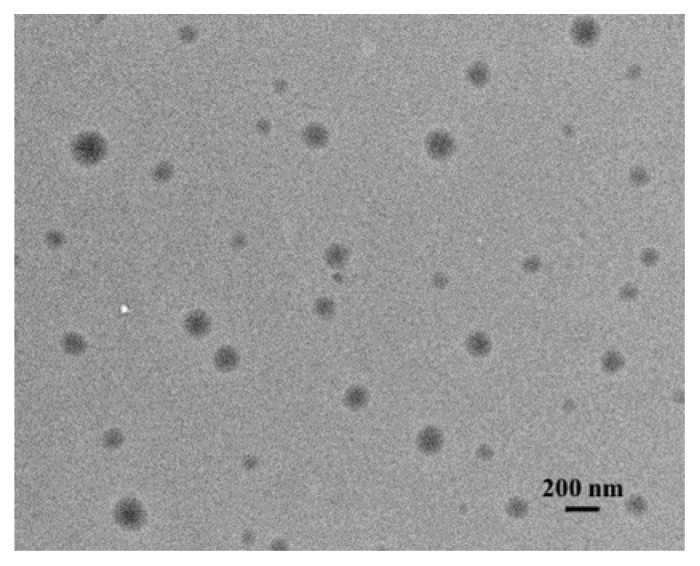
Micrograph obtained on a transmission electron microscope for PC/Tw20/C_14_PB at molar ratio of components 1/0.2/0.025.

The shape and size of the transfersomes was also confirmed by transmission electron microscopy (TEM) (Figure 2 and Figure S1).

The ultimate goal of this study is the transdermal delivery of 2-PAM, and the evaluation of its effect on the physicochemical characteristics of transfersomes was of key importance. According to the results presented in Table 2, 2-PAM had no effect on the hydrodynamic diameter and the PdI compared to empty vesicles. However, there is a clear decrease in the absolute value of the zeta potential of transfersomes loaded with 2-PAM. Such values do not contribute to the colloidal stability of nanocontainers, since a minimum of 30 mV of the surface charge of liposomes is necessary to prevent aggregation with subsequent destruction of vesicles [25]. However, probably due to storage conditions (4 °C), the studied systems did not show any signs of destruction. It should be noted that the PC/Tw20 system with 2-PAM appears to be a less stable system. This is evidenced by an increase in the hydrodynamic diameter and the PdI for transfersomes loaded with 2-PAM (139 ± 2 nm, 0.242 ± 0.006) compared with empty vesicles (106 ± 1 nm, 0.057 ± 0.014) after 1 month of storage. This assumption is also based on the fact that empty transfersomes showed acceptable physicochemical parameters after a year of storage (Table S1), while for transfersomes with 2-PAM, it was not possible to obtain the correct values of the hydrodynamic diameter and the zeta potential, which probably indicates that they were collapsed.

According to Table 2, transfersomes modified with C_14_PB have a greater ability to encapsulate the substrate and have an advantage over unmodified transfersomes in this characteristic. It should be noted that an increase in the concentration of C_14_PB leads to an insignificant increase in encapsulation efficiency (EE%).

### 2.2. In Vitro/Ex Vivo Release Study

**Figure 3:**
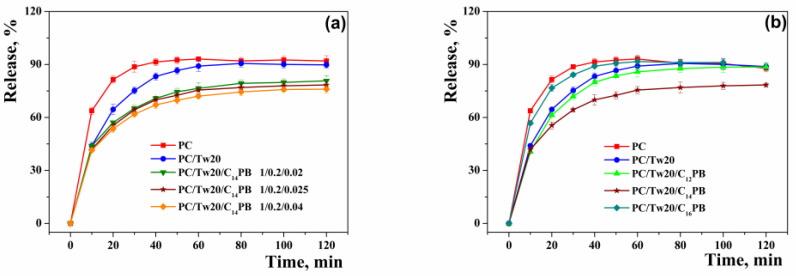
In vitro 2-PAM release from transfersomes, modified with: (**a**) C_14_PB at different molar ratio of components; (**b**) C_n_PB at constant molar ratio of components 1/0.2/0.025, phosphate buffer (0.025 M), pH = 7.4, 37 °C.

In vitro release of 2-PAM was performed using the classical method—dialysis. The study was carried out in two directions: (1) by varying the concentration of C_14_PB (Figure 3a); (2) by varying the lengths of the hydrocarbon tail of the C_n_PB (Figure 3b). It is clear that the addition of a surfactant (Tw20 or C_n_PB) slows down the release of 2-PAM from the vesicles. This phenomenon is more pronounced for C_n_PB. While the differences in the release profiles are insignificant in the case of varying the concentration of C_14_PB, the release curves change significantly in the case of varying the hydrocarbon tail length. The prolongation of 2-PAM release is expressed in the following order: C_14_PB > C_12_PB > C_16_PB. It should be noted that 2-PAM was released very quickly, and by the second hour of the experiment, almost all of it was detected outside the dialysis bag. The absorption spectra of 2-PAM released during dialysis are shown in Figures S2 and S3.

**Figure 4:**
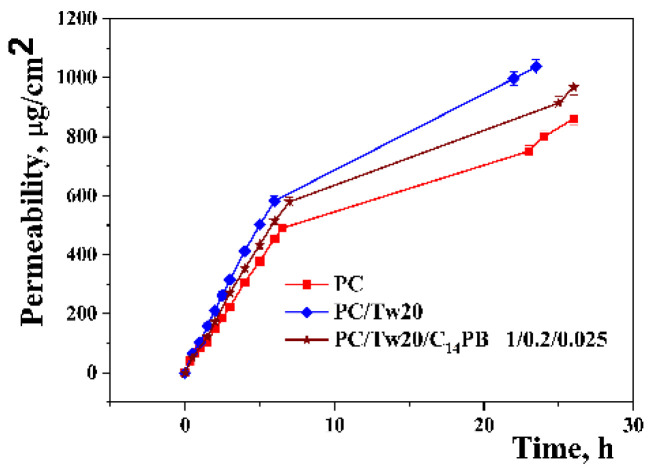
Ex vivo penetration of transfersomes with 2-PAM (μg/cm^2^), phosphate buffer (0.025 M), pH = 7.4, 34 °C.

To investigate the influence of vesicular systems on 2-PAM permeation, the skin of Wistar rats was placed on Franz diffusion cells. This experiment allows for preliminary assessment of the effect of the studied surfactants on the properties of vesicles, in particular, on the penetrating ability ex vivo. According to Figure 4, the inclusion of a surfactant in the composition of vesicles increases the concentration of 2-PAM that penetrates through the rat skin compared to conventional liposomes. It is worth pointing out that, contrary to expectations, the addition of a C_14_PB to the lipid bilayer resulted in a slower penetration of 2-PAM through the skin.

Comparing the in vitro and ex vivo results, it can be seen that the trend in the prolongation of 2-PAM release from unmodified and modified transfersomes persists: modification of transfersomes with C_14_PB results in a slower release of 2-PAM. The absorption spectra of 2-PAM released using Franz cells are shown in Figure S4.

### 2.3. In Vitro Toxicity Assessment

**Figure 5:**
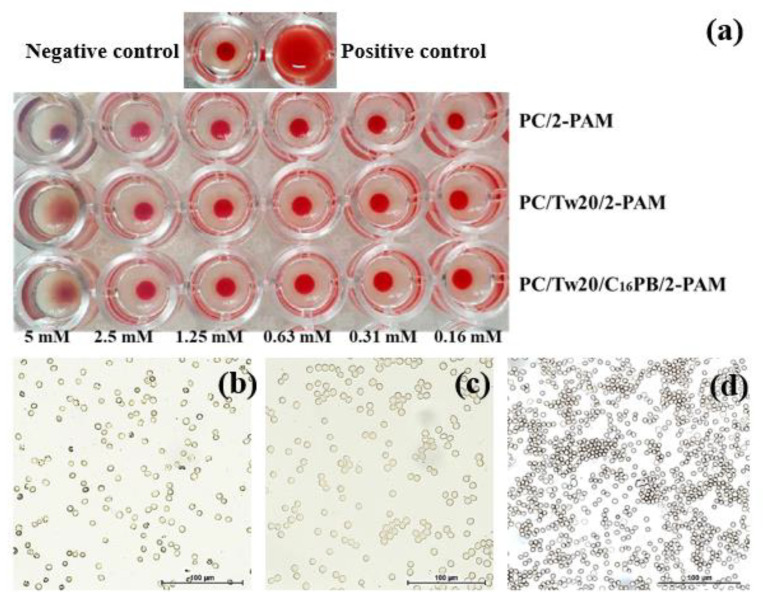
(**a**) Photograph of microplate wells containing blood samples with addition of unmodified and modified transfersomes. Agglutination of erythrocytes observed using transmitted light microscopy with the addition of: (**b**) PC/Tw20/2-PAM; (**c**) PC/Tw20/C_16_PB/2-PAM 1/0.2/0.025; (**d**) positive control of agglutination.

Transfersome toxicity was assessed in three directions: hemolysis, hemagglutination, and cytotoxicity. The results of the hemagglutination activity assessment are shown in Figure 5. In the control wells containing a solution of erythrocytes in saline, a tight button of erythrocytes (dense layer at the bottom of the well) was observed, indicating a negative reaction. Agglutinated cells forming a carpet at the bottom of the well were observed in the positive control (in mixture of type A (II) and B (III) erythrocytes).

The obtained results demonstrated that agglutination was observed for systems PC/Tw20/2-PAM and PC/Tw20/C_16_PB/2-PAM only at the highest concentrations (Figure 5a). A decrease in the concentration of the studied samples showed the absence of agglutination, which was confirmed by bright field microscopy data (Figure 5a,b). Conventional liposomes did not cause agglutination at all tested concentrations. Additionally, all studied systems exhibited low hemolytic activity, even at high concentrations (Table S2). However, it should be noted that C_14_PB-modified transfersomes show lower hemolytic activity than other homologues.

Transfersome cytotoxicity was evaluated using cervical epithelial carcinoma cell line (M-HeLa) and normal lung cells (WI-38). Table 3 shows the concentrations of transfersome dispersions (relative to PC), at which 50% of cells die (IC_50_). It becomes obvious that the inclusion of surfactants in the composition of liposomes increases their cytotoxic effect relative to both cancerous and normal cells. However, modification of transfersomes with cationic C_n_PB leads to a more selective action, which is more pronounced for systems with C_14_PB.

### 2.4. Evaluation of Effectiveness In Vivo

To determine the level of AChE reactivation in blood, several forms of 2-PAM were tested in vivo (Table 4). First, rats were poisoned with a sub-lethal dose of paraoxon (POX) (0.6 mg/kg). An hour later, a gel form of free and transfersomal 2-PAM was applied to the depilated skin area on rat backs. Blood from the tail vein was taken at regular time intervals to determine the level of AChE reactivation over the time after application of the gels. The percentage of AChE reactivation was determined from the average level of inhibited AChE 1 h after POX administration (52.27 ± 2.37%). According to Table 4, the gel form of PC/Tw20 showed statistically significant (23.65 ± 7.23%, *p* = 0.005) level of AChE reactivation after 6 h of application, then PC/Tw20/C_14_PB by 6 h of the experiment. Free 2-PAM was practically not active.

Since OP poisoning is fatal without timely treatment, an experiment was conducted on the survival of rats with different treatment strategies. Based on the results of AChE reactivation, it was decided to keep the gel forms on the rat skin for 24 h. Further POX (1 mg/kg) was injected and the number of surviving animals was estimated. According to the results presented in Table 5, the classical method of treating OP poisoning (2-PAM intravenously) is only 55% effective, while preliminary transdermal treatment of the skin with a gel form of transfersomal 2-PAM increases the survival rate of rats up to 90%.

The final step in evaluating the effectiveness of vesicular systems in vivo was the study of the pharmacokinetics of 2-PAM in the blood of rats. At this stage, only one leading system (PC/Tw20) was selected. To assess the pharmacokinetics of 2-PAM in the blood, three time points were chosen: 6, 12 and 24 h. The maximum concentration of 2-PAM in the blood is reached by 6 h, after which a plateau is observed (Figure S5). This confirms the uniform flow of the drug into the bloodstream, which is typical for transdermal drug administration.

## 3. Discussion

Phosphatidylcholine and Tween 20 are classic transfersomal ingredients [26,27,28,29,30]. In addition to Tween 20, other non-ionic surfactants can also be included in the transfersome composition: sodium deoxycholate, sorbitan monooleate (Span 80), polysorbate 80 (Tween 80) [31,32,33], etc. However, there are almost no references in the literature of a combination of non-ionic and cationic surfactants included in transfersomes, but there are studies in which cationic lipids were used [34]. An increase in the zeta potential of lipid nanocontainers is a key factor in their stability over time and in increasing drug flux through the skin. However, from this point of view, it is necessary to maintain a certain balance between the toxicity of cationic systems and the positive effect. In the present work, an attempt was made to increase the efficiency of classical transfersomes by incorporating cationic surfactants with a pyrrolidinium head group with different hydrocarbon tail lengths (C_n_PB). This combination is new and requires careful analysis of the effect of C_n_PB on the physicochemical properties of transfersomes.

Due to transfersome extrusion through a polycarbonate membrane with a pore size of 100 nm, the hydrodynamic diameter of the systems was maintained at 100–120 nm (Table 1). It should be noted that the addition of a non-ionic surfactant to liposomes led to a decrease in the transfersome size, probably due to the formation of mixed micelles, which leads to a decrease in phospholipid monomers and compaction of aggregates [30,35,36]. According to the results, the size of the vesicles slightly increased with increasing concentration of C_n_PB compared with classical transfersomes, and approached the size of PC liposomes. This is probably due to the repulsive force inside the interbilayers of vesicles [25]. This fact was confirmed by an increase in the zeta potential of transfersomes due to an increase in C_n_PB monomers in the lipid bilayer. In general, the size of all systems was in the nanometer range, which was also confirmed by the TEM (Figure 2 and Figure S1). It can be seen from the photographs that the shape of the aggregates was spherical.

An important characteristic of liposomal systems is stability over time. Within 2.5 months, the zeta potential of transfersomes decreased. Table 1 shows that there is a slight increase in the hydrodynamic diameter of the transfersomes during storage (no more than 6%). According to the literature data, liposomal systems are characterized by a slight increase in size and polydispersity index during storage [37,38,39,40]. It is worth noting that changes in size in most cases were insignificant; however, it is mentioned that fresh liposomes show greater efficiency than liposomes stored for a long time [41]. Taking this into account, freshly prepared transfersomes were used for all in vitro, ex vivo and in vivo experiments. It had also been shown that the greater stability of liposomes depends on the storage conditions, namely the storage temperature [42]. As in the present study, 4 °C is the most preferred temperature as it eliminates thermal effects on the vesicles and the lipid bilayer remains more rigid [43,44]. The strongest increase in size and PdI was observed for the PC/Tw20 system. The zeta potential of this system became close to neutral, which probably led to slight sticking of the transfersomes with each other. Interestingly, with further monitoring of stability, most of the systems were stable for more than a year (Table S1). It should be noted that the aforementioned trend changes after a year, and the size, on the contrary, becomes smaller with an increase in the C_14_PB concentration in the bilayer. In case of such long storage, it is obvious that systems with higher charge are likely to be more stable.

The incorporation of oximes into nanoparticles based on human serum albumin [45,46,47,48], transferrin-modified mesoporous silica nanoparticles [49], liposomes [50,51,52], solid-lipid nanoparticles [53,54,55], and polymer nanoparticles [56,57] has been reported. There are only a few examples of successful in vivo experiments with AChE reactivators in nanocontainers [50,51,53,54,55,57]. However, research on transdermal delivery of 2-PAM is a new direction, which can reduce mortality due to poisoning with OPs by increasing the effectiveness of traditional treatment. The classic way to study lipid nanocarriers loaded with a substrate is to determine the aggregate size, encapsulation efficiency, and substrate release rate. According to the results presented in Table 2, the EE% of all systems was higher than 50%. However, modification of transfersomes with C_14_PB made it possible to increase EE% up to >60%. For the dodecyl and hexadecyl homologues the EE% values are slightly lower. This may be due to the ability of surfactants to increase the size of transfersomes and increase the hydrophilic core of vesicles. The dodecyl homologue, due to the length of the hydrocarbon tail, probably integrates worse, while the hexadecyl homologue strongly loosens the lipid bilayer and leads to leakage of the substrate. This was supported by in vitro 2-PAM release curves (Figure 3). For the hexadecyl derivative, the release of 2-PAM was much faster compared to C_12_PB and C_14_PB (Figure 3b).

The assessment of percutaneous permeation of drugs is a key step in the evaluation of dermal or transdermal delivery systems. Among rodents, rat skin is the most structurally similar to human skin and is the most commonly used model. However, some studies report that rat skin is generally more permeable than human skin [58], but the pattern of penetration of a particular system can be traced. Figure 4 shows the release curves of 2-PAM included in transfersomes through the skin of Wistar rats. As can be seen, the incorporation of surfactants into the lipid bilayer slowed down the penetration of 2-PAM through the skin, as was in the case with the release of the substrate in vitro. From the point of view of the use of transdermal formulations to prevent poisoning of the organism with OPs during prolonged contact, the prolongation of the action of modified transfersomes is a positive effect.

Evaluation of the toxicity of any nanoscale drug delivery systems is a particularly important aspect of research. The blood compatibility test performed for different concentrations of transfersomes (Table S2). The studied systems practically did not show hemolytic activity in the entire concentration range. The shapes of the erythrocytes were found to be normal, and no deformity, swelling and shrinkage was observed (Figure 5). Erythrocyte hemagglutination was tested for selected systems, in particular for transfersomes modified only with C_16_PB. This is due to the fact that the toxicity of surfactants often increases with the length of the hydrocarbon tail, and the absence of hemagglutination activity in the hexadecyl homologue may indicate the absence of toxicity for C_12_PB and C_14_PB [59,60]. It should be noted that the inclusion of cationic surfactants in the composition of transfersomes increased the cytotoxic effect of the systems toward cancer cell line M-HeLa. Cytotoxicity toward the normal WI-38 cell line also increased, but a more selective effect was observed for the PC/Tw20/C_14_PB system (Table 3).

To further evaluate the in vivo effectiveness of transfersomes, a gel form of vesicles was developed using Carbopol^®^ 940. The pH of the gel form of transfersomes was maintained at the level of 5–5.5. This is due to the fact that the pH of the skin lies in the range of 4.2 and 5.6. The closer the pH of the drug to this range, the lower the degree of ionization, therefore, the permeability can be improved and the irritant effect on the skin can be eliminated [61,62]. To determine the degree of AChE reactivation, four groups of Wistar rats were used (Table 4). According to the presented results, statistically significant level of AChE reactivation was reached only for unmodified transfersomes by 6 h (23.65 ± 7.23%) and 24 h (24.2 ± 9.73%) of treatment.

After transfersomal 2-PAM was shown to be effective in reactivating AChE in the blood, the survival of rats poisoned with POX was evaluated. According to the results obtained, only transdermal administration of 2-PAM was insufficient to achieve the expected effect in the traditional treatment of OP poisoning (intravenous administration of 2-PAM) (Table 5). These results support the concept of prevention of OP poisoning developed in this work: the prophylactic administration of transfersomal 2-PAM through the skin in combination with the traditional treatment method increased the survival rate of rats up to 90% compared to 55%, when only emergency post-exposure therapy was used.

For a more detailed understanding of the drug fate in the blood, plasma pharmacokinetic analysis was performed. The analysis was carried out for the PC/Tw20 system. After 6 h, the maximum concentration of 2-PAM in the blood was 35.9 ± 5.8 ng/mL. Further, after 12 and 24 h, there is a slight decrease in the concentration of the drug to 21.5 ± 3.3 ng/mL and 20.6 ± 2.9 ng/mL, respectively (Figure S5). This allowed to maintain a certain level of drug in the blood for up to 24 h, which is important for the prophylactic use of 2-PAM in the treatment of acute OP poisoning.

## 4. Materials and Methods

### 4.1. Materials

L-α-Phosphatidylcholine (soybean, 95%) (Avanti Polar Lipids, Inc., Alabaster, AL, USA), Tween 20 (Ferak Berlin GmbH, Berlin, Germany) and pyridine-2-aldoxime methochloride (Sigma-Aldrich, St. Louis, USA) were used as received. Pyrrolidinium surfactants (C_n_PB, where *n* = 12, 14, 16) were synthesized as previously described [24]. Carbopol^®^ 940 were purchased from Acros Organics (New Jersey, NJ, USA). Transfersomal dispersions were prepared using ultrapure Milli-Q water (Direct-Q5 system, Millipore SAS, Molsheim, France).

### 4.2. Animals

All experiments with animals were carried out in accordance with the Directive of the Council of the European Union 2010/63/EU. The protocol of experiments was approved by the Animal Care and Use Committee of FRC Kazan Scientific Center of RAS. Animals were kept in a well-ventilated room at 20–22 °C in a 12 h light/dark cycle, 60–70% relative humidity. The rats were not limited in food and water. Wistar rats were purchased from the Laboratory Animal Breeding Facility (Branch of Shemyakin-Ovchinnikov Institute of Bioorganic Chemistry, Puschino, Moscow Region, Russia).

### 4.3. Vesicle Preparation

Thin lipid film hydration method was utilized for production of conventional and substrate-loaded transfersomal dispersions. PC (10 mM), Tw20 (2 mM) and C_n_PB (0.2 mM, 0.25 and 0.4 mM depending on the ratio of components) were mixed in 100–200 µL of chloroform until all solids are completely dissolved. The volume of the organic solvent was selected depending on the total mass of the components, i.e., on the final volume of the transfersomal dispersions. For transfersomal dispersions up to 2 mL, the components were dissolved in 100 µL of an organic solvent, and for systems up to 10 mL, the components were dissolved in 200 µL of an organic solvent. The organic phase was evaporated at 40–45 °C for 30 min at 40 rpm using a RE-52AA rotary evaporator (Shanghai Jingke Scientific Instrument Co., Ltd., Shanghai, China). The next step was hydration of thin lipid film, followed by incubation of the solutions in a water bath (60 min at 60 °C). The drug-free transfersomes were hydrated with Milli-Q water. To load the drug into transfersomes, the lipid film was hydrated with an aqueous solution of 2-PAM. To achieve a high degree of monodispersity of the systems, transfersomes were subjected to a freeze/thaw cycle (5 times using liquid nitrogen) and extrusion through polycarbonate membranes with a pore size of 100 nm using LiposoFast Basic extruder (Avestin, OT, Canada). Large volumes of transfersome dispersion were obtained using an automatic LiposoFast LF-50 extruder (Avestin, Ottawa, Canada). For all experiments, each system was obtained at least 3 times to confirm the reproducibility of the results.

### 4.4. Particle Zeta Potential and Size Distribution Analysis

The hydrodynamic diameter (D_h_), the polydispersity index (PdI), and the zeta potential (ζ) of transfersomes were measured using dynamic and electrophoretic light scattering on a Zetasizer Nano ZS device (Malvern Instruments Ltd., Worcestershire, UK). Transfersome dispersions were diluted with Milli-Q water to a total lipid concentration of 2 mM and placed in a disposable folded capillary cell (DTS1070, Malvern Instruments Ltd., Worcestershire, UK) for measurements. During measurements, the sample was exposed to a Helium–Neon laser with a power of 4 mV and a wavelength of 633 nm. In the experiment, back scattered light at an angle of 173° was detected. The effective hydrodynamic radius (R_h_) and the zeta potential were calculated according to the Einstein–Stokes Equation (1) and Smoluchowski Equation (2), respectively:

D = k_B_T/6πηR_h_ (1)

where D is the diffusion coefficient, k_B_ is the Boltzmann’s constant, T is the absolute temperature, η is the solvent viscosity, and R_h_ is hydrodynamic radius;

ζ = μη/ε(2)

where ζ is the zeta potential, η is the dynamic viscosity of the fluid, μ is the particle mobility and ε is the dielectric constant.

### 4.5. Transmission Electron Microscopy (TEM)

TEM images were obtained at the Interdisciplinary Center for Analytical Microscopy of Kazan (Volga Region) Federal University, using a Hitachi HT7700 Exalens microscope, (Hitachi, Tokyo, Japan). The images were acquired at an accelerating voltage of 100 kV. Samples were dispersed on 300 mesh 3 mm copper grids (Ted Pella) with continuous carbon-formvar support films. To exclude transfersome damage, 5 μL of the solution was dried at room temperature.

### 4.6. Potentiometry

The pH of the gel form of transfersomes was determined using a pH-211 pH meter (Hanna Instruments, Woonsocket, Rhode Island, USA) with an accuracy of 0.05 pH units. Before each experiment the glass electrode was calibrated according to standard solutions.

### 4.7. Drug Loading and Quantification of Encapsulation Efficiency (EE%)

Since 2-PAM is a hydrophilic drug, the lipid film was hydrated with its aqueous solution (5 mg/mL) for efficient loading. The separation of the non-encapsulated substrate from the encapsulated one was carried out as previously described using Amicon^®^ Ultra-15 Centrifugal Filter Units (Merck Millipore, Burlington, MA, USA) [59]. To determine the concentration of unencapsulated 2-PAM, 5 μL of 2-PAM solution from the bottom of the centrifuge filters was diluted to 4 mL and the optical density was determined spectrophotometrically in a 1 cm quartz cuvette (Specord 250 Plus, Analytik Jena AG, Jena, Germany). The absorption maximum of 2-PAM is at 294 nm, and the extinction coefficient is 11,962 M^−1^∙cm^−1^. The 2-PAM concentration was calculated using the Bouguer–Beer–Lambert law. To calculate the drug encapsulation efficiency, the following equation was used:

EE% = (Total amount of 2-PAM–free 2-PAM)/(Total amount of 2-PAM) × 100%(3)

### 4.8. In Vitro Substrate Release Rate Analysis

The release rate of 2-PAM from transfersomes was determined in vitro using dialysis bags with a pore size of 3.5 kDa (Scienova GmbH, Jena, Germany). Dialysis bags with 0.5 mL transfersomal dispersion were immersed in 200 mL sodium phosphate buffer (0.025 M, pH = 7.4). The experiments were carried out at 37 °C with constant stirring (200 rpm). To determine the amount of released substrate, the optical density of 2-PAM in the external medium was determined in a 1 cm quartz cuvette at fixed time intervals using Specord 250 Plus (Analytik Jena AG, Jena, Germany). The studies were terminated at the stop of the optical density increase.

### 4.9. In Vitro Toxicity Assessment

Transfersome toxicity was assessed against human erythrocytes and against tumor and normal cell lines. Hemolytic and hemagglutination activity of transfersomes was assessed using human erythrocyte mass (Group IV). Determination of hemagglutination activity was carried out exactly according to the previously described method [63]. Red blood cell adhesion could be observed both with the unaided eye and under a Nikon Eclipse Ci-S microscope (Nikon, Nanjing, China).

The degree of hemolysis was determined by comparing the optical density of hemoglobin released into the solution at 100% hemolysis and the optical density after treatment of the erythrocyte mass with transfersomes [64]. The degree of hemolysis was determined in a wide range of concentrations of transfersome dispersions.

The cytotoxicity of transfersomes toward M-HeLa (epithelioid cervical carcinoma) and WI-38 (human lung fibroblasts) cell lines was estimated by means of the multifunctional Cytell Cell Imaging system (GE Health Care Life Science, Uppsala, Sweden) using the Cell Viability Bio App. Cell lines were purchased from the Type Culture Collection of the Institute of Cytology (Russian Academy of Sciences). The cells were cultured in a standard Eagle’s nutrient medium and supplemented with 10% fetal calf serum and 1% non-essential amino acids, which were then plated into a 96-well plate (Eppendorf) at a concentration of 10^5^ cells/mL, and cultured in a CO_2_ incubator at 37 °C. Cells were treated with the transfersomal systems after 24 h of cultivation at concentration of 150 μL per well. DAPI (4′,6-diamidin-2-phenylindol, Sigma-Aldrich, St. Louis, USA) and propidium iodide (Sigma-Aldrich, St. Louis, MO, USA) were used to dye living and dead cells, respectively to calculate living and dead cells from the fluorescence intensity of the corresponding fluorescent probes. Intact cells cultured were used as a control.

### 4.10. Ex Vivo Substrate Release Studies

Before in vivo experiments, several systems were tested for their ability to pass through the skin ex vivo. The studies were carried out using Franz cells (SES GmbH Analysesysteme, Bechenheim, Germany). The characteristics of Franz cells and a detailed description of the experiment were published earlier in [65]. Before the experiment, the skin was soaked in phosphate buffer (pH = 7.4) for 30 min. Franz cells were filled with phosphate buffer (C = 0.025 M, pH = 7.4), where 2-PAM was released from transfersomes that passed through the skin of rats. The optical density of 2-PAM in receptor cell was measured at fixed time intervals using 0.1 cm quartz cuvette on Specord 250 Plus (Analytik Jena AG, Jena, Germany).

### 4.11. AChE Reactivation in Rat Blood

To assess the reactivation degree of AChE in blood, a gel form of transfersomes was prepared as follows: Carbopol^®^ 940 was dissolved in Milli-Q water at a concentration of 2% *w*/*v* and stirred at 500 rpm for 5 h. Transfersomes loaded with 2-PAM (10 mg/mL) were mixed with Carbopol^®^ 940 in a ratio of 1:1 (*w*/*w*) and stirred until a homogeneous mass was obtained. Triethanolamine was used as a pH adjustment agent. 2 h prior to gel application the hair on the back of rats (2 × 2 cm) was first shaved and then depilated by hair remover cream. The gel form of transfersomes (0.6 g) was applied on shaved areas 1 h after the administration of POX (0.6 mg/kg). For the study, 5 groups of animals were selected, and each group included 5 rats: (1) non-poisoned control; (2) poisoned with POX; (3) poisoned with POX and received free 2-PAM; (4) poisoned with POX and received unmodified transfersomes with 2-PAM; (5) poisoned with POX and received modified transfersomes with 2-PAM. Blood samples were taken from the tail vein of rats before POX injection, 1 h after POX injection and then 2, 4, 6, 24 h after applying the gel to the rat skin. The volume of blood was 200 μL at each sampling. Blood was mixed with 50 ME of heparin per 1 mL of blood, centrifuged at 8000 rpm for 8 min at 4 °C using Eppendorf 5430R centrifuge with FA-45-30-11 rotor (Eppendorf AG, Hamburg, Germany), and then erythrocytes were collected. Hemolysis of erythrocytes was induced by diluting the blood 100 times with a 0.03% solution of Triton X-100 in phosphate buffer (0.1 M, pH = 8.0). The hemolyzed sample was incubated with 5 × 10^−5^ M iso OMPA for 20 min. AChE activity was assessed spectrophotometrically using PerkinElmer λ25 spectrophotometer (PerkinElmer Inc., Waltham, MA, USA) by measuring the color intensity at a wavelength of 436 nm at 25 °C [66]. The reaction mixture contained 400 µL of hemolyzed erythrocytes, 20 µL of 0.01 M Ellman’s reagent and 1560 µL of 0.1 M phosphate buffer (pH = 8.0). The mixture was incubated for 10 min at 25 °C to achieve complete reaction of the matrix sulfhydryl groups with Ellman’s reagent before adding the substrate. The enzymatic reaction was started by adding 20 μL of 0.1 M acetylthiocholine and the rate of reaction was measured for two min. The percentage of AChE reactivation was expressed as the mean ± the standard error.

### 4.12. 2-PAM Pharmacokinetics in Rat Plasma

The pharmacokinetics of 2-PAM in the blood of Wistar rats was studied for unmodified transfersomes after transdermal administration. A gel form of transfersomes (0.6 g) was applied to the shaved areas (2 × 2 cm) on the backs of rats. Blood was taken at 6, 12 and 24 h after transfersome application. Rats were first deeply anesthetized by isoflurane inhalation. Blood samples were collected and mixed with 50 ME of heparin per 1 mL of blood and centrifuged at 8000 rpm for 8 min at 4 °C using Eppendorf 5430R centrifuge with FA-45-30-11 rotor (Eppendorf AG, Hamburg, Germany) to get plasma. There were 6 rats at each time point. The 2-PAM extraction procedure from plasma is described in [51]. The calibration curves were linear in the range 10.0–300.0 ng/mL (Figure S6).

### 4.13. Rat Survival 

To test the Wistar rats’ survival, 3 different treatment strategies for POX poisoning were tested: (1) rats were poisoned with POX without any therapy; (2) 0.6 g of the gel form of unmodified transfersomes was applied for 24 h, then rats were poisoned with POX; (3) rats were poisoned with POX, then treated 10 min post-exposure with free 2-PAM; (4) 0.6 g of the gel form of unmodified transfersomes was applied for 24 h, then rats were poisoned with POX and treated 10 min post-exposure with free 2-PAM. In all groups of animals POX was intraperitoneally administered at the dose of 1 mg/kg. Free 2-PAM was intravenously administered at the dose of 10 mg/kg. Stabulated animals were observed during 72 h after injection of POX.

### 4.14. Data Analysis and Statistics

All data processing was performed using Microsoft Excel 2016^®^ and OriginPro 8.5. Result significance was determined in the IBM SPSS Statistics program, version 22.0. Statistical analysis was performed using the Mann–Whitney test. *p* < 0.05 was considered statistically significant.

## 5. Conclusions

Thus, for the first time, a gel containing 2-PAM in the transfersomal form was obtained for transdermal application as a preventative countermeasure against OP poisoning. For this purpose, stable classical and modified transfersomes were obtained and characterized in the absence and presence of 2-PAM in terms of the hydrodynamic diameter, the zeta potential, and encapsulation efficiency. Modification of transfersomes with amphiphiles results in sustained release of 2-PAM from vesicles. The combination of results on hemolysis and hemagglutination allows us to consider these systems as safe to be introduced in the bloodstream. It has been shown that unmodified and modified transfersomes are able to increase the level of reactivated AChE in vivo compared with free 2-PAM by up to 23% and 11%, respectively. Pre-exposure transdermal administration of gel containing classical transfersomes with 2-PAM (24 h before POX poisoning) in combination with post-exposure intravenous administration of free 2-PAM leads to an increase in the survival rate of rats from 55% to 90%.

## Figures and Tables

**Table 1:** Physicochemical properties of transfersomes, modified by C_n_PB at different molar ratio of components.

System	Molar Ratio	D_h_, nm	PdI	ζ, mV	D_h_, nm	PdI	ζ, mV
		1 Day			2.5 Months		
PC	-	113 ± 1	0.086 ± 0.009	−14 ± 1	118 ± 1	0.119 ± 0.043	−11 ± 1
PC/Tw20	1/0.2	107 ± 1	0.057 ± 0.002	−11 ± 1	153 ± 13	0.232 ± 0.003	−1 ± 1
PC/Tw20/C_14_PB	1/0.2/0.02	111 ± 1	0.072 ± 0.019	21 ± 1	115 ± 1	0.071 ± 0.005	15 ± 1
PC/Tw20/C_14_PB	1/0.2/0.025	117 ± 2	0.084 ± 0.004	29 ± 1	121 ± 1	0.075 ± 0.006	20 ± 1
PC/Tw20/C_14_PB	1/0.2/0.04	120 ± 2	0.096 ± 0.003	38 ± 1	126 ± 1	0.083 ± 0.014	23 ± 1
PC/Tw20/C_12_PB	1/0.2/0.025	110 ± 2	0.089 ± 0.015	23 ± 1	112 ± 1	0.076 ± 0.019	17 ± 1
PC/Tw20/C_16_PB	1/0.2/0.025	116 ± 2	0.101 ± 0.004	24 ± 1	121 ± 1	0.093 ± 0.004	22 ± 1

**Table 2:** Physicochemical properties of modified transfersomes with 2-PAM at different length of the surfactant hydrocarbon tail and ratio of components.

System	Molar Ratio	EE, %	D_h_, nm	PdI	ζ, mV	D_h_, nm	PdI	ζ, mV
			1 Day			1 Month		
PC	-	60.3 ± 2.8	118 ± 1	0.082 ± 0.012	−2 ± 0.5	116 ± 2	0.115 ± 0.004	−4 ± 1
PC/Tw20	1/0.2	55.3 ± 0.9	110 ± 1	0.061 ± 0.010	−2 ± 0.2	139 ± 2	0.242 ± 0.006	−3 ± 1
PC/Tw20/C_14_PB	1/0.2/0.02	63.5 ± 3.3	105 ± 1	0.041 ± 0.009	12 ± 1	118 ± 1	0.111 ± 0.009	3 ± 1
PC/Tw20/C_14_PB	1/0.2/0.025	64.7 ± 5.3	107 ± 2	0.061 ± 0.014	17 ± 1	103 ± 1	0.047 ± 0.017	5 ± 1
PC/Tw20/C_14_PB	1/0.2/0.04	66.2 ± 2.1	114 ± 2	0.078 ± 0.009	24 ± 1	121 ± 2	0.099 ± 0.012	13 ± 1
PC/Tw20/C_12_PB	1/0.2/0.025	56.9 ± 0.1	110 ± 1	0.076 ± 0.010	10 ± 1	110 ± 1	0.143 ± 0.020	6 ± 1
PC/Tw20/C_16_PB	1/0.2/0.025	56.1 ± 1.5	117 ± 1	0.074 ± 0.016	9 ± 1	104 ± 1	0.085 ± 0.001	8 ± 1

**Table 3:** The cytotoxic effect of transfersomes, loaded with 2-PAM, toward normal (WI-38) and cancer (M-HeLa) human cell lines.

System	Molar Ratio	IC_50_, mM *	
		M-HeLa	WI-38
PC	-	3.60 ± 0.28	>5
PC/Tw20	1/0.2	1.68 ± 0.13	1.64 ± 0.12
PC/Tw20/C_12_PB	1/0.2/0.025	0.48 ± 0.04	1.25 ± 0.10
PC/Tw20/C_14_PB	1/0.2/0.025	0.51 ± 0.04	1.44 ± 0.11
PC/Tw20/C_16_PB	1/0.2/0.025	0.75 ± 0.06	1.00 ± 0.08

* Concentrations are presented relative to PC. The experiments were repeated for three times. The results are expressed as the mean ± standard deviation (SD).

**Table 4:** AChE reactivation profile after transdermal administration of transfersomes loaded with 2-PAM.

Blood Sampling Time after Applying, h	Group of Treatment			
	POX, 1 h, *n* = 5	POX, 1 h, + free 2- PAM, *n* = 5	POX, 1 h, + PC/Tw20/2-PAM, *n* = 5	POX, 1 h, + PC/Tw20/C_14_PB/2-PAM 1/0.2/0.025, *n* = 5
	AChE reactivation in rat erythrocytes, %			
2	1.66 ± 3.79 *p* = 0.940	1.35 ± 3.40 *p* = 0.706	1.36 ± 5.57 *p* = 1.000	8.40 ± 7.52 *p* = 0.449
4	1.80 ± 3.74 *p* = 0.571	1.46 ± 5.95 *p* = 1.000	1.41 ± 5.20 *p* = 0.821	8.73 ± 7.18 *p* = 0.304
6	0.62 ± 3.16 *p* = 0.735	4.46 ± 9.30 *p* = 0.483	23.65 ± 7.23 *p* = 0.005 *	11.98 ± 4.06 *p* = 0.05
24	2.85 ± 7.39 *p* = 0.678	7.25 ± 6.13 *p* = 0.404	24.2 ± 9.73 *p* = 0.021 *	20.10 ± 11.80 *p* = 0.113

* Significantly different compare to POX poisoned group without therapy, *p* < 0.05 (Mann–Whitney test).

**Table 5:** Survival of rats poisoned with POX after different treatment strategies.

Group of Treatment	*n*/N *	% of Surviving Rats
POX	0/20	0%
POX + 2-PAM intravenously 10 min after POX	11/20	55%
PC/Tw20/2-PAM transdermally for 24 h + POX	8/20	40%
PC/Tw20/2-PAM transdermally for 24 h + POX + 2-PAM intravenously 10 min after POX	18/20	90%

* *n*—the number of surviving animals in a group; N—total animals in the group.

## Data Availability

Analyzed data are included in this manuscript. Raw data are available from the authors upon request.

## Supplementary Materials

The supporting information can be downloaded at: https://www.mdpi.com/article/10.3390/ijms232314992/s1.
