# Supplementary material for: Transdermal Delivery of 2-PAM as a Tool to Increase the Effectiveness of Traditional Treatment of Organophosphate Poisoning
Source: Int J Mol Sci. 2022 Nov 30;23(23):14992. doi: 10.3390/ijms232314992 (PMC9735786; doi:10.3390/ijms232314992)
Supplement: Supplementary file 1 [file ijms-23-14992-s001.zip › ijms-1954520-supplementary.pdf]

Article

Transdermal Delivery of 2-PAM as a Tool to Increase the Effectiveness of Traditional Treatment of Organophosphate Poisoning

Leysan Vasileva, Gulnara Gaynanova, Irina Zueva, Anna Lyubina, Syumbelya Amerhanova, Daina Buzyurova, Vasily Babaev, Alexandra Voloshina, Konstantin Petrov and Lucia Zakharova *

Arbuzov Institute of Organic and Physical Chemistry, FRC Kazan Scientific Center of RAS, 8 Arbuzov str., 420088, Kazan, Russian Federation

Fax: (843) 273 18 72. E-mail: luciaz@mail.ru

Table S1. Physicochemical properties of transfersomes, modified by C_nPB at different molar ratio of components.

System	Molar ratio	D_h, nm	PdI	ζ, mV
		12 months		
PC	-	Unstable		
PC/Tw20	1/0.2	187±1	0.426±0.028	-14±1
PC/Tw20/C_{14}PB	1/0.2/0.02	150±2	0.222±0.004	17±1
PC/Tw20/C_{14}PB	1/0.2/0.025	133±2	0.203±0.012	20±1
PC/Tw20/C_{14}PB	1/0.2/0.04	120±2	0.054±0.016	40±1
PC/Tw20/C_{12}PB	1/0.2/0.025	133±2	0.213±0.004	18±2
PC/Tw20/C_{16}PB	1/0.2/0.025	123±1	0.131±0.006	29±1

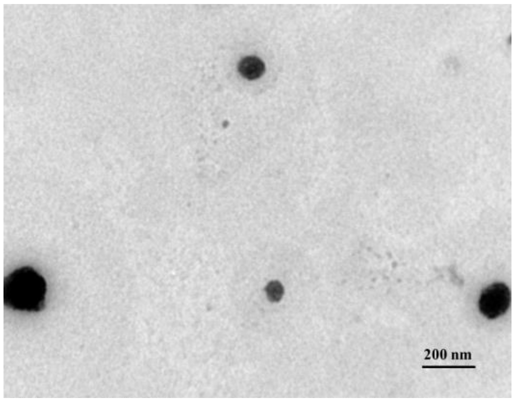

Figure S1. Micrograph obtained on a transmission electron microscope for PC/Tw20.

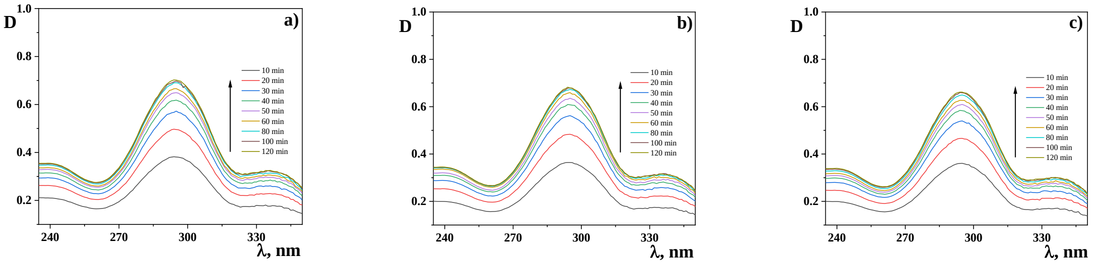

Figure S2. The absorption spectra of 2-PAM at different time intervals of release by dialysis for PC/Tw20/C_{14}PB at different molar ratio of components: a) 1/0.2/0.02; b) 1/0.2/0.025; c) 1/0.2/0.04, phosphate buffer (0.025 M), pH=7.4, 37°C; the arrow indicates the direction of dialysis duration increasing.

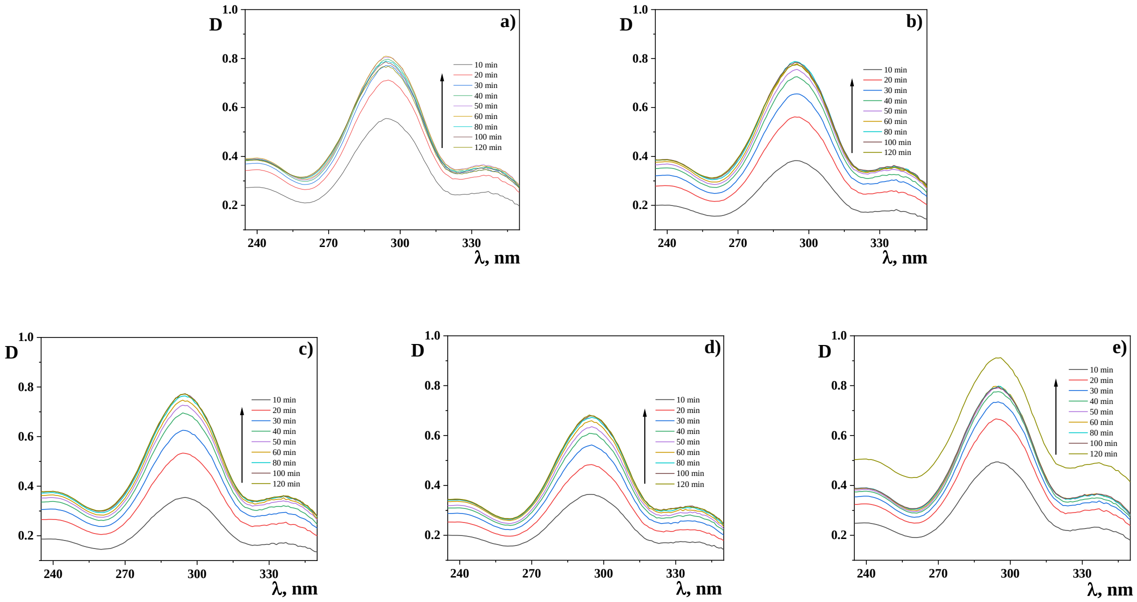

Figure S3. The absorption spectra of 2-PAM at different time intervals of release by dialysis for: a) PC; b) PC/Tw20; c) PC/Tw20/C_{12}PB 1/0.2/0.025; d) PC/Tw20/C_{14}PB 1/0.2/0.025; e) PC/Tw20/C_{16}PB 1/0.2/0.025, phosphate buffer (0.025 M), pH=7.4, 37°C; the arrow indicates the direction of dialysis duration increasing.

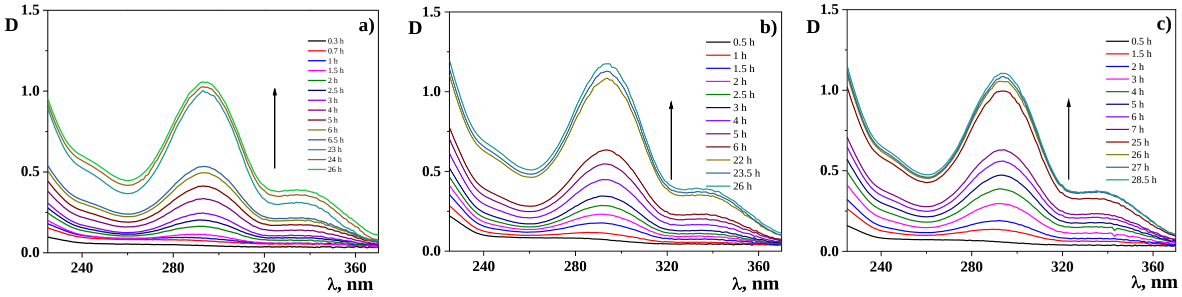

Figure S4. The absorption spectra of 2-PAM at different time intervals of release on Franz cells for aqueous form of vesicles *ex vivo*: a) PC; b) PC; b) PC/Tw20; c) PC/Tw20/C_{14}PB at molar ratio of components 1/0.2/0.025, phosphate buffer (0.025 M), pH=7.4, 34°C; the arrow indicates the direction of dialysis duration increasing.

Table S2. Hemolytic activity of transfersomes, loaded with 2-PAM at molar ratio of components 1/0.2/0.025.

**Tested compounds**	**Hemolysis, %**				
	**Concentration of PC, mM**				
	5.0	2.5	1.25	0.63	0.31
PC	16.6	9.9	4.6	2.2	0.0
PC/Tw20	15.8	8.2	3.7	1.7	0.0
PC/Tw20/C_{12}PB	20.0	7.9	3.8	1.4	0.0
PC/Tw20/C_{14}PB	15.9	7.0	3.8	1.8	0.0
PC/Tw20/C_{16}PB	18.7	7.2	5.5	2.2	0.0

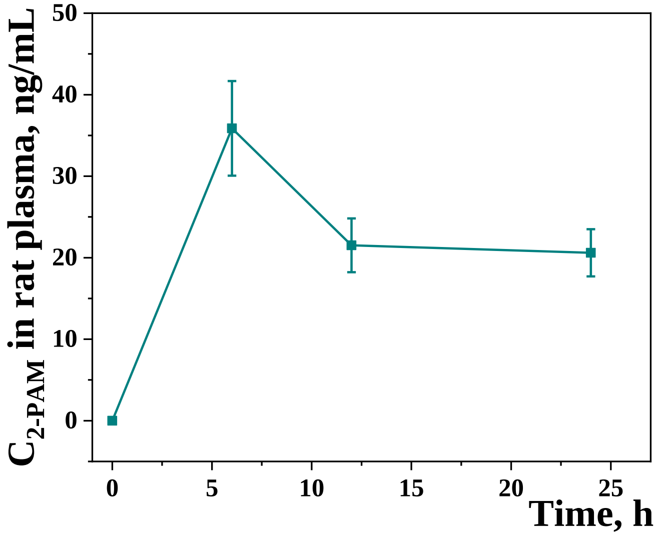

Figure S5. Plasma concentration of 2-PAM after transdermal administration of 2-PAM via PC/Tw20 transfersomes determined by HPLC-ESI-MS. Data are presented as the mean ± standard deviation for 5 rats.

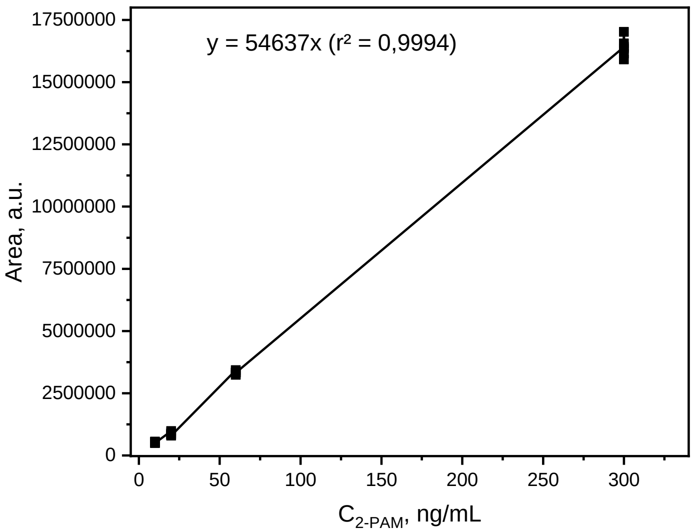

Figure S6. Calibration curve of 2-PAM in rat plasma.
